# Gossypiboma after Breast Augmentation

**DOI:** 10.1155/2013/808624

**Published:** 2013-02-14

**Authors:** Kira Lundin, Julie E. Allen, Lene Birk-Soerensen

**Affiliations:** Department of Plastic Surgery, Aalborg University Hospital, Soendre Skovvej 3, 9000 Aalborg, Denmark

## Abstract

A 39-year-old woman was referred for removal of cosmetic breast implants and related siliconoma. After an exchange of breast implants at a private clinic a year previously, she had asymmetry of the right breast, persistent pain, and a generally unacceptable cosmetic result. An MRI had shown a well-defined area with spots of silicone-like material at the upper pole of the right breast. Surgical removal of presumed silicone-imbibed breast tissue was undertaken, and surprisingly a gossypiboma was found in its place, which had not been identified on the MRI. Gossypiboma is the condition of an accidentally retained surgical sponge. This complication is also known as a textiloma, gauzoma, or muslinoma and is well described in other surgical specialties. However, it is extremely rare after plastic surgery, and this case illustrates the need for continued attention to the surgical count of sponges and instruments.

## 1. Case

A 39-year-old woman was referred for removal of breast implants because of persistent pain and an unacceptable cosmetic result. The patient had a history of thrombophilia but was otherwise healthy and had a BMI within normal range.

Twelve months before admission to our department, the patient had had breast implants changed at a private clinic, apparently because of implant rupture. The original augmentation was performed 9 years ago for cosmetic reasons. After the second procedure, the patient suffered from pain and intermittent swelling as well as a hard consistency and change of contour of the right breast.

**Figure 1:**
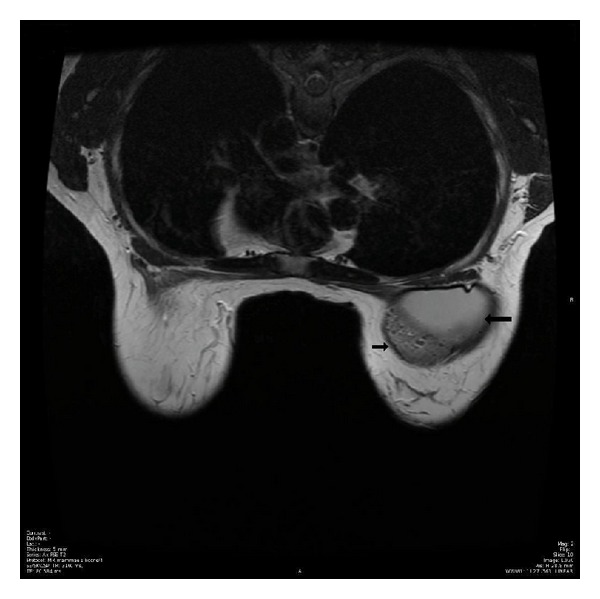
MRI scan showing the gossypiboma (small arrow) as a well-defined area dotted with silicone-like material above the breast implant (large arrow) on the right side, corresponding to the area where the patient felt a painful lump. The implant in the left breast is not visible in this slide.

An ultrasound and mammography had showen no sign of malignant disease. An MRI scan 6 months after the last operation showed a well-defined area dotted with small spots of silicone-like material as seen in Figure 1. It was concluded that the assumed silicone could be remnants from the original breast implants which had been exchanged for new.

The patient was referred to our department by her local GP, and an indication was found for removal of both implants in the public healthcare system, as the private clinic had recently closed.

**Figure 2:**
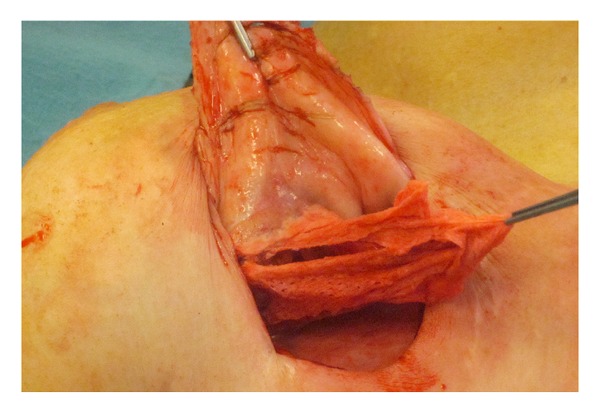
Perioperative photograph of retained surgical sponge embedded in the fibrous capsule in the upper pole of the right breast.

During surgery, both implants were removed and found to be intact. The irregular lump was believed to be silicone-imbibed breast tissue due to a previous implant rupture that was lodged in the capsule, and upon dissection of this, a retained surgical sponge was identified, as shown in Figure 2. There was no apparent radioopaque marker in the sponge.

The postoperative course was uneventful and the patient's symptoms of discomfort disappeared within 2 weeks. It is not known whether a surgical count of sponges and instruments was performed during the patient's second operation one year previously.

## 2. Discussion

The term gossypiboma is derived from the Latin word “gossypium” meaning cotton. The condition is also known as a retained surgical sponge, a textiloma, gauzoma, or muslinoma. The incidence of gossypiboma has been described as 1 : 5000–1 : 19.000 procedures in other surgical specialties but is likely to be significantly underreported [1, 2]. Gossypibomas related to plastic surgery procedures appear to be extremely rare and have only been described a few times previously [3–7].

Adherence to guidelines regarding surgical count of sponges and instruments is mandatory, and continued vigilance must be observed. It is the duty of both the surgeon and the assisting nurse to ensure a correct surgical count. In the regrettable event of a retained foreign object after surgery, it is important to secure documentation and to be of assistance with respect to the medicolegal aspects. We would recommend that all surgical sponges contain a radio-opaque marker to facilitate identification.
